# Molecular and biological characterization of some circulating strains of Newcastle disease virus in broiler chickens from Eastern Saudi Arabia in 2012-2014

**DOI:** 10.14202/vetworld.2019.1668-1676

**Published:** 2019-10-30

**Authors:** Abdullah I. A. Almubarak

**Affiliations:** Department of Microbiology, College of Veterinary Medicine, King Faisal University, Saudi Arabia.

**Keywords:** broiler chickens, Newcastle disease virus, Saudi Arabia

## Abstract

**Background and Aim:**

Newcastle disease (ND) is a worldwide poultry disease that is historically known to cause severe losses in the poultry industry. In the present study, attempts were made to characterize ND virus (NDV) recovered from broiler chickens in the Eastern Region of Saudi Arabia from January 2012 to March 2014.

**Materials and Methods:**

Reverse transcription-polymerase chain reaction was used for the detection of NDV followed by partial sequencing of the fusion (F) gene. The intracerebral pathogenicity index (ICPI), mean death time (MDT), and complete sequencing of the hemagglutinin-neuraminidase (HN) gene were also used for further biological and molecular characterization.

**Results:**

NDV was detected at a rate of 9.6% (11/115) of the tested flocks, most of which were vaccinated against ND. F gene-based phylogeny and motifs of the fusion protein cleavage site (FPCS) showed segregation of Saudi isolates into two groups. The first group contained 10 isolates and was located in genotype II with the lentogenic motif ^112^GRQGRL^117^ at the FPCS. The second group contained one isolate and was located in genotype VII with velogenic motif ^112^RRQKRF^117^. Further characterization using the ICPI and MDT of two representative isolates showed virulence of both tested isolates. Phylogenetic analysis of the HN gene showed close nucleotide identity between the two isolates. A BLAST search for sequences similar to HN gene sequences showed high identity with isolates from the surrounding region.

**Conclusion:**

The present findings showed a low detection rate of NDV, possibly due to the wide application of vaccines, and the circulation of at least two NDV genotypes, II and VII, in the Eastern Region of Saudi Arabia. The present Saudi isolates may share common ancestors with isolates from the surrounding region.

## Introduction

Newcastle disease (ND) is one of the most important poultry diseases in terms of the number of birds lost [1]. The causative agent, ND virus (NDV), is an avian paramyxovirus type I (APMV-1) belonging to the genus *Avulavirus* and family *Paramyxoviridae* [2]. It has the ability to infect a wide range of avian species, and 240 bird species were reported to be susceptible [3,4]. Wild and domesticated birds were implicated as a reservoir of NDV and a possible mode of dissemination [5,6]. According to the World Organization for Animal Health (OIE), the presence of multiple basic amino acids at the fusion protein cleavage site (FPCS) and/or an intracerebral pathogenicity index (ICPI) of ≥0.7 is required to designate APMV-1 as the cause of an ND outbreak [7]. The mean death time (MDT) is also frequently used to classify APMV-1 as velogenic (MDT <60 h), mesogenic (MDT 60-90 h), and lentogenic (MDT >90 h) [8]. Phylogenetically, NDV has been genotyped into two major clades: Class I and Class II. Class I is divided into nine genotypes (1-9) and is mostly composed of avirulent viruses for chickens. Class II viruses occur in at least 18 genotypes (I-XVIII) and include most virulent as well as some avirulent and vaccine strains [9-11]. Alternatively, these genotypes are rearranged in six lineages (1-6). Genotypes I, II, VI, and VII are renamed lineages 1, 2, 4, and 5, respectively. Genotypes III, IV, V, and VIII are grouped into a single lineage called lineage 3 [12]. The sixth lineage includes viruses with high antigenic and genetic divergence from APMV-1 and viruses that belong to Class I [8,12]. Due to the confusion ensuing from the simultaneous use of these two systems, Diel *et al*. proposed a unified nomenclature in which Class I is renamed genotype 1, 10 genotypes of Class II retain their names (genotypes I-IX and XI), and five new genotypes are added, genotypes X, XII, XIII, XIV, and XV [13].

In the Middle East, ND outbreaks have occurred since the late 1960s [14]. ND records in Saudi Arabia go back to the 1980s when velogenic NDV strains were reported by El-Zein [15]. Isolates from Saudi Arabia were typed as lineages 4b, 4c, and 5d (genotypes VI and VII) based on the fusion (F) gene phylogeny [12]. Isolates belonging to genotype VIId were also recently reported [16]. Poultry in Saudi Arabia has experienced high mortality rates in recent years [17]. Among the affected regions is the Eastern Region of Saudi Arabia, which was estimated to produce 37,598,000 broiler chickens and 1,197,306,000 eggs in 2017 [18].

To elucidate the role played by NDV in these mortalities, a survey targeting this virus was launched in the Eastern Region of Saudi Arabia. Herein, we report the characterization of circulating NDV in this region from 2012 to 2014.

## Materials and Methods

### Ethical approval

All experimental procedures and management conditions used in this study were approved by the Ethics Committee at King Faisal University, Saudi Arabia.

### Study design

Tissue samples were collected from broiler chickens in the Eastern Region of Saudi Arabia. Nucleic acid was extracted and NDV was detected using reverse transcription-polymerase chain reaction (RT-PCR). Part of the F gene, including the region encoding FPCS, was sequenced to genotype the detected virus. NDV was isolated in embryonated specific-pathogen-free (SPF) eggs to perform *in vivo* biotyping. Two representative isolates were selected to carry out the more aggressive procedure, ICPI analysis. In addition, MDT analysis and sequencing of the complete hemagglutinin-neuraminidase (HN) gene were also performed for the two representative isolates.

### Sample collection and processing

Tissue samples, including those from the trachea and lung, were collected from broiler chickens in the Eastern Region (Al-Hasa and Dammam) of Saudi Arabia from January 2012 to March 2014. Purposive sampling was used to collect samples from broilers with signs of respiratory illness such as nasal discharge, rales, and gasping. Samples were collected from commercial poultry farms, poultry slaughterhouses, and poultry clinic at the Veterinary Teaching Hospital of King Faisal University. Attempts were made to collect complete history for sampled flocks. All samples were collected from commercial farms with several sheds on each farm. The number of chicks per shed varied from 10,000 to 15,000 chicks. Biosecurity measures were applied at the majority of the farms and included minimizing the entrance of visitors and vehicles and, when visitors and vehicles were allowed to enter, disinfecting the vehicles and having visitors wear special clothes and boots. Visiting other farms and the transport of litter, equipment or birds between farms were not permitted. Dead birds and litter were disposed of as directed by veterinarians. Measures were also taken to avoid the entrance of wild animals and the transmission of infection from hatcheries. The farms were separated by approximately 10 km or more. For each flock, tissue samples from each organ from all sampled birds were pooled together and treated as a single sample. Samples were collected in 10 volumes of phosphate-buffered saline (PBS) containing gentamicin and nystatin, both at a concentration of 50 µg/ml and stored at −80°C until homogenization. Homogenization was performed with the Biospec Mini-Beadbeater and the Omni International Ceramic Beads kit. An IQeasy Plus Viral DNA/RNA Extraction kit (Cat # 17153, iNtRON Biotechnology, South Korea) was used according to the manufacturer’s instructions to extract viral RNA from homogenized tissues. Extracted RNA was stored at −80°C until use to produce cDNA by the Reverse Transcription System (Cat # A3500, Promega, USA) according to the manufacturer’s instructions.

### Detection and genotyping of NDV

Detection and genotyping of NDV were performed using nested PCR as previously described by Nanthakumar *et al*. [19]. Primers were obtained from Integrated DNA Technologies (IDT, Coralville, IA). For detection, GoTaq^®^ Green Master Mix (Cat # M7122, Promega) was used in a final volume of 25 µl that contained primers at a final concentration of 0.8 µM. For genotyping, the reaction was repeated with positive samples using the i-StarMAX II Master Mix (Cat # 25174, iNtRON Biotechnology) at a final volume of 50 µl with a final primer concentration of 0.2 µM. Similarly, PCR targeting the HN gene was also used for genotyping as previously described by Tan *et al*. [20]. PCR products were purified using the Wizard SV Gel and PCR Clean-up System (Cat # A1460, Promega) according to the manufacturer’s instructions. Purified amplicons were sequenced by Macrogen Sequencing Service (South Korea).

### Sequence analysis

Sequence analysis was performed using Molecular Evolutionary Genetics Analysis (MEGA) X software [21]. Sequences were aligned using ClustalW. A phylogenetic tree was constructed using the maximum likelihood method with a bootstrap value of 1000 replicates. A BLAST search was performed to determine the most related sequences in GenBank. F gene-based phylogenic classification of Diel *et al*. [13] was followed to prepare the phylogenetic tree. Reference sequences representing the genotypes and subgenotypes in the Diel classification were used. Genotype XV, which was previously reported to harbor recombinant strains, was not included [22-24]. For the HN gene-based phylogeny, reference sequences from a similar study of the region [20] were used, as were the most similar sequences found in BLAST analysis. Sequence identity is the number of identical nucleotides between pairs of sequences divided by the total length of aligned sequences.

### GenBank accession number

Sequences obtained from the present 11 isolates of NDV were deposited in GenBank with the following accession numbers: MK608020-MK608024 and MK660772-MK660777 for F gene sequences and MK660778 and MK660779 for HN gene sequences, as shown in Table-1.

**Table 1:** Data on the NDV-positive samples.

Sample ID	Date of collection	Age (days)	Number of sampled birds	Governorate	NDV vaccine-age of vaccination	Detected NDV genotype	GB # of F gene
NDV-SA/Chicken/NH3	April 23, 2012	30	20	Dammam	B1-1 day; LaSota-18 days	Avirulent genotype II	MK660776
NDV-SA/Chicken/NH10	March 5, 2013	14	5	Hasa	B1-1 day	Virulent genotype II	MK660772
NDV-SA/Chicken/NH12	March 12, 2013	21	8	Hasa	Clone 30-1 day; Clone 30-14 days	Virulent genotype II	MK660773
NDV-SA/Chicken/NH17	November 27, 2013	46	>20	Hasa	B1-1 day; Clone 30-20 days	Virulent genotype VII	MK608022*
NDV-SA/Chicken/NH22	December 15, 2013	31	>20	Hasa	Clone-1 day; Clone 30-20 days	Virulent genotype II	MK660774
NDV-SA/Chicken/NH24	December 17, 2013	11	>20	Hasa	B1-1 day	Virulent genotype II	MK608023*
NDV-SA/Chicken/NH25	December 29, 2013	34	>20	Dammam	B1-1 day; Clone 30-20 days	Virulent genotype II	MK608024
NDV-SA/Chicken/NC3	February 10, 2013	34	1	Hasa	B1-1 day; LaSota-16 days	Avirulent genotype II	MK608020
NDV-SA/Chicken/NC19	May 27, 2013	37	1	Hasa	B1-1 day; LaSota-20 days	Avirulent genotype II	MK660777
NDV-SA/Chicken/NC37	2013	NA	1	Hasa	NA	Avirulent genotype II	MK660775
NDV-SA/Chicken/NC60	2013	NA	1	Hasa	NA	Virulent genotype II	MK608021

NA=Data were not available, GB#=GenBank accession number

* MK660779 and MK660778 are the GenBank accession numbers for the HN gene of NDV-SA/Chicken/NH17 and NDV-SA/Chicken/NH24, respectively. The sample pairs NDV-SA/Chicken/NH3 with NDV-SA/Chicken/NH25, NDV-SA/Chicken/NH12 with NDV-SA/Chicken/NC3, and NDV-SA/Chicken/NH17 with NDV-SA/Chicken/NH24 were collected from the same farms but different periods, flocks, and sheds. NDV=Newcastle disease virus

### Isolation

Isolation was carried out in SPF embryonated eggs (Nile SPF, Egypt) according to Alexander [25]. Before inoculation, homogenized tissue samples were centrifuged at 1000× g for 10 min. The centrifugation-derived supernatant was passed through a 0.2 µm sterile nylon syringe filter (Thermo Scientific, Nalgene^®^, Cat #195-2520, USA). A 200 µl volume of filtrated liquid was inoculated into the allantoic cavity in 10-day-old embryonated eggs. Embryonic death during the first 24 h was considered non-specific. Thereafter, embryonic deaths were considered as indicators of the presence of NDV, and allantoic fluids (AFs) were harvested.

### MDT

MDT was measured following the method described by Grimes [26]. Ten-fold serial dilutions (10^−1^-10^−9^) of the AFs were prepared in sterile PBS. Each dilution was inoculated into five 10-day-old SPF embryonated eggs (Nile SPF). One hundred microliters were inoculated in the allantoic cavity. Eggs were then sealed with paraffin wax and incubated at 37°C. Eggs were candled every 8 h for 7 days. The highest virus dilution that induced the death of all inoculated embryos was considered the minimal lethal dose, and the meantime (hours) required for this dilution to kill inoculated embryos was considered the MDT.

### ICPI

ICPI analysis was carried out according to OIE [7]. SPF chicks (Nile SPF) aged 30-40 h were used for this test. Briefly, fresh AF with hemagglutination (HA) titer of 2^5^ was diluted in sterile normal saline (1/10), and 50 µl was injected intracerebrally into 10 chicks for each sample. Chicks were observed daily for 8 days and scored 0 if normal, 1 if sick, and 2 if dead. The index was calculated as the mean score per bird per observation over the 8 days.

## Results and Discussion

### NDV detection

During the study period, a total of 115 flocks were sampled. Samples were tested for the presence of NDV using RT-PCR. The results showed that 11 (9.6%) samples were positive for NDV. Table-1 presents the data on the NDV-positive samples. This value is comparable to the previously reported 10% NDV prevalence in Bangladesh [27]. Higher prevalence of 41.6% in Jordan [28], 60% in Ghana [29], and 62% in Egypt [30] were reported. In all of these cases, diseased flocks were targeted; however, a much lower prevalence is expected when samples are collected regardless of disease status, as in Brazil, where 0.7% was reported [31].

### Partial F gene-based phylogeny and FPCS

**Figure-1:**
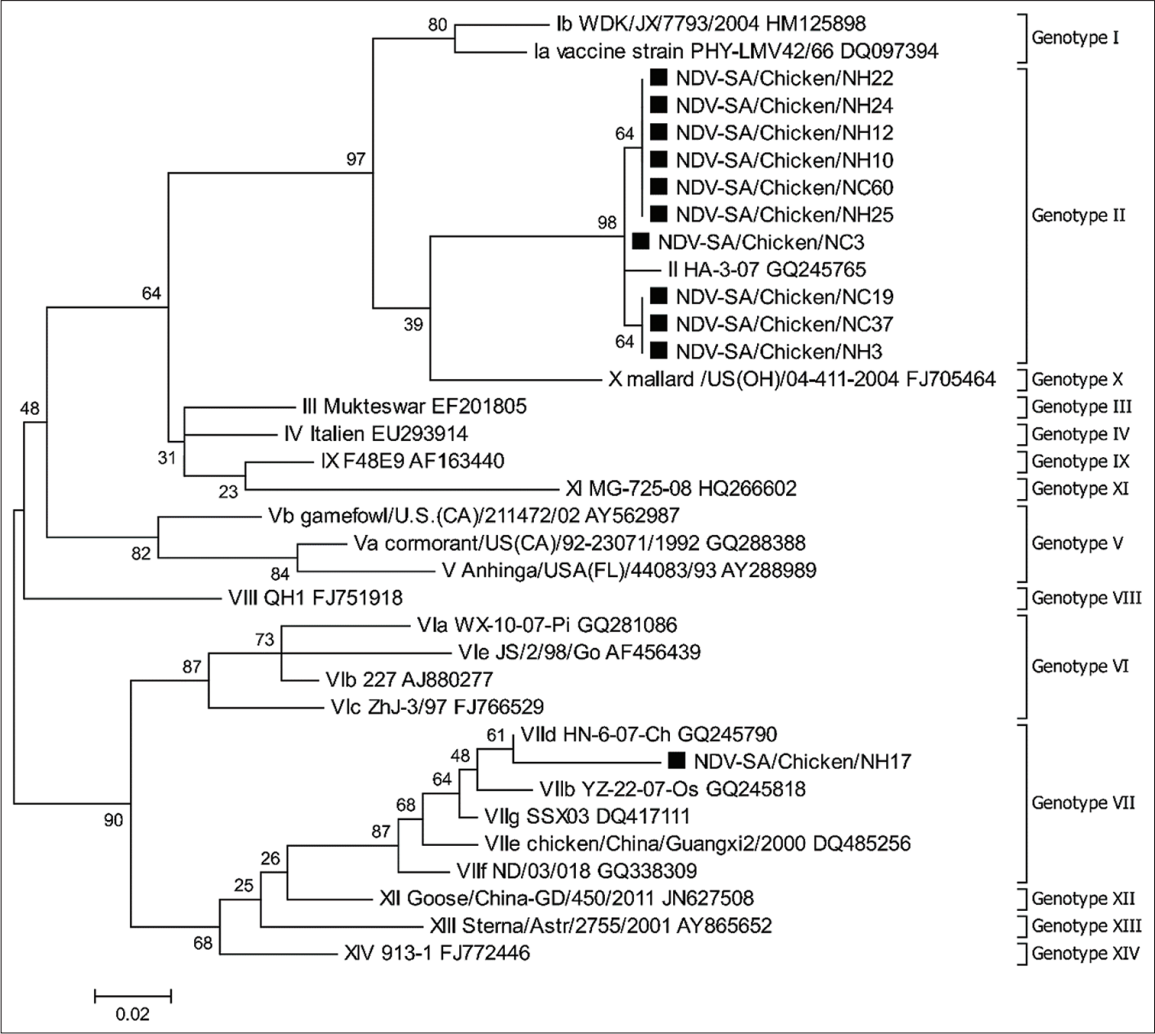
Partial F gene-based phylogeny showing the relationship between Saudi Newcastle disease virus isolates and reference sequences. The present Saudi isolates are marked with black squares. Reference sequences end with their GenBank accession numbers.

According to the classification of Diel *et al*. [13], the present 11 NDV isolates segregated into two genotypes (Figure-1). The first group was located within genotype II with 99-100% nucleotide identity to each other. The second group contained only one isolate that clustered with reference sequences from genotype VII. The deduced amino acid sequences of the FPCS showed two motif types compatible with the results of the F gene phylogeny. The 10 isolates in genotype II revealed the lentogenic motif ^112^GRQGRL^117^ at the FPCS. The single isolate in genotype VII showed the velogenic motif ^112^RRQKRF^117^ at the FPCS. This is in agreement with results from the Eastern Region of Saudi Arabia recently reported by Al-Ali *et al*. [16], where genotype VII was detected during the years 2015/2016.

Isolates belonging to genotype II may originate from vaccine strains. The commonly used live attenuated vaccines, including LaSota, Hitchner-B1, Clone 30, and VG/GA strains, all belong to genotype II [32]. On the other hand, genotype VII has been reported in Eastern Asia since 1985 and has been divided into two subgenotypes, VIIa and VIIb. The former includes viruses that spread to Europe and Asia, while the latter includes viruses that spread to South Africa. Subsequently, additional subgenotypes were added, including VIIc, VIId, and VIIe containing isolates from China, Kazakhstan, and South Africa and VIIf, VIIg, and VIIh consisting of African isolates [9]. Collectively, genotype VII became the most prevalent NDV genotype in Asia and Europe and was incriminated in causing the fourth NDV panzootic that started in Southeast Asia in the early 1990s**.** Within this genotype, subtype VIId, to which the present Saudi isolate belongs, appears to be the most predominant subtype [33-37].

### Isolation and BLAST search

A BLAST search for sequences similar to the partial F gene sequences yielded a result compatible with observations from NDV isolation. Isolation in SPF embryonated eggs revealed that seven isolates were able to induce embryonic death in inoculated eggs during the 2^nd^ and 3^rd^ days post-inoculation. The remaining four isolates of genotype II were not able to induce embryonic death in inoculated eggs. A BLAST search for the 10 isolates revealed that Chinese strain HA-3-07-Ch and LaSota vaccine (GB#: GQ245765; AF077761) are the most similar sequences in GenBank, with 98-99% nucleotide identity. A BLAST search for the NDV-SA/Chicken/NH17 isolate of genotype VII revealed that the strain most closely related to this isolate was the NDV/Chicken/2/SA/2016 (GB# MG022112), with 97.6% nucleotide identity. Based on the available overlapped sequence (167 nt), 96.4% nucleotide identity was also found with the previously reported genotype VII Saudi isolate ISACK00184 (GB # AY135754) that was isolated in 2000. Comparing the present Saudi NDV sequences with the three most similar reference sequences revealed the nucleotide substitutions presented in Table-2.

**Table 2:** Comparison of the present Saudi sequences with the three most similar sequences in BLAST over the sequenced region of the F gene.

Nt position in the F gene according to GB# M24693	GenBank accession #	286	289	292	298	347	375	418	430	439	458	463	481	484	490
Genotype VII															
NDV-SA/Chicken/NH17	MK608022	G	G	-	C	-	A	C	T	G	-	A	T	A	T
NDV/Chicken/2/SA/16	MG022112	A	A	-	T	-	A	C	T	G	-	A	C	A	C
NDV-SA-1SACK00184/00	AY135754	A	A	-	T	-	G	T	G	G	?	?	?	?	?
NDV-China/Chicken/HN-6-07-Ch	GQ245790	A	G	-	T	-	A	T	G	A	-	G	C	G	T
Genotype II															
NDV-SA/Chicken/NH24*	MK608023	G	-	G	-	G	-	-	-	-	G	-	T	-	-
NDV-SA/Chicken/NH3**	MK660776	G	-	G	-	A	-	-	-	-	C	-	T	-	-
NDV-SA/Chicken/NC3	MK608020	G	-	G	-	A	-	-	-	-	G	-	T	-	-
NDV-B1	AF309418	C	-	T	-	A	-	-	-	-	G	-	A	-	-
NDV-LaSota	AF077761	C	-	T	-	A	-	-	-	-	G	-	A	-	-
NDV-HA-3-07-Ch/07	GQ245765	C	-	T	-	A	-	-	-	-	G	-	T	-	-

-=identical nucleotide; ?=sequence not available.

* Sequences NDV-SA/Chicken/NH10, NDV-SA/Chicken/NH12, NDV-SA/Chicken/NH22, NDV-SA/Chicken/NH25, and NDV-SA/Chicken/NC60 showed complete identities with the NDV-SA/Chicken/NH24 sequence.

** Sequences NDV-SA/Chicken/NC19 and NDV-SA/Chicken/NC37 showed complete identities with the NDV-SA/Chicken/NH3 sequence. NDV=Newcastle disease virus

### *In vivo* biotyping by ICPI and MDT analyses

Two Saudi isolates, NDV-SA/Chicken/NH17 and NDV-SA/Chicken/NH24, representing genotype VII and genotype II, respectively, were selected for further characterization by ICPI and MDT analyses. Both isolates showed a high ICPI: 1.54 in the case of NDV-SA/Chicken/NH17 and 1.6 in the case of NDV-SA/Chicken/NH24. Similarly, the MDT was measured for both isolates and was 52 h for NDV-SA/Chicken/NH24 and 57 h for NDV-SA/Chicken/NH17. Tables-3 and 4 summarize the results of the molecular and biological characterizations and the clinical signs associated with the present NDV isolates.

**Table 3:** Summary of the molecular and biological characterization of the Saudi NDV isolates.

Isolate	Molecular characterization				Biological characterization		
	
	F gene-based genotyping	* F gene BLAST (GB #)	FPCS (112-117)	HN gene-based genotyping	Lethality to embryonated eggs	MDT (h)	ICPI
NDV-SA/Chicken/NH3	II	AF077761	GRQGRL	--	-	--	--
NDV-SA/Chicken/NH10	II	GQ245765	GRQGRL	--	+	--	--
NDV-SA/Chicken/NH12	II	GQ245765	GRQGRL	--	+	--	--
NDV-SA/Chicken/NH17	VII	MG022112	RRQKRF	VII	+	57	1.54
NDV-SA/Chicken/NH22	II	GQ245765	GRQGRL	--	+	--	--
NDV-SA/Chicken/NH24	II	GQ245765	GRQGRL	VII	+	52	1.6
NDV-SA/Chicken/NH25	II	GQ245765	GRQGRL	--	+	--	--
NDV-SA/Chicken/NC3	II	AF077761	GRQGRL	--	-	--	--
NDV-SA/Chicken/NC19	II	AF077761	GRQGRL	--	-	--	--
NDV-SA/Chicken/NC37	II	AF077761	GRQGRL	--	-	--	--
NDV-SA/Chicken/NC60	II	GQ245765	GRQGRL	--	+	--	--

-- not determined;

* GenBank accession number of most similar sequence in BLAST. NDV=Newcastle disease virus, HN=Hemagglutinin-neuraminidase, FPCS=Fusion protein cleavage site, ICPI=Intracerebral pathogenicity index, MDT=Mean death time

**Table 4:** Relevant clinical data on groups of Saudi NDV isolates according to their molecular and biological characteristics.

Molecular/biological group	Genotype II, not lethal to embryonated eggs	Genotype II, lethal to embryonated eggs	Genotype VII	Ambiguous isolate (genotypes II/VII)
Isolates	NDV-SA/Chicken/NH3, NDV-SA/Chicken/NC3, NDV-SA/Chicken/NC19 NDV-SA/Chicken/NC37*	NDV-SA/Chicken/NH10, NDV-SA/Chicken/NH12, NDV-SA/Chicken/NH22, NDV-SA/Chicken/NH25, NDV-SA/Chicken/NC60*	NDV-SA/Chicken/NH17	NDV-SA/Chicken/NH24
Clinical picture	In general, there were mild respiratory signs, nasal discharge, and rales	Unthriftiness, depression, nasal discharge, gasping, and rales	Many birds showed depression, nasal discharge, gasping, coughing, rales, and conjunctivitis	Similar to that of NDV-SA/Chicken/NH17
Mortality/1000 chicks in the day preceding sample collection	5 chicks in the source of isolate NDV-SA/Chicken/NC19	20 chicks in the source of isolate NDV-SA/Chicken/NH22 and 35 chicks in the source of isolate NDV-SA/Chicken/NH10	40 chicks	21 chicks
Postmortem examination	Congestion and exudate in trachea	Congestion and exudate in trachea and congestion of intestine. In some birds, congestion and enlargement of kidneys were seen	Congestion of trachea, intestine, and kidney and enlargement of kidney and spleen. Purulent pneumonia was seen in some birds	Congestion of trachea and intestine, exudate in tracheas, and enlargement of spleen

* Farms separated by approximately 10 km or more. NDV=Newcastle disease virus

### Complete HN gene-based phylogeny

**Figure-2:**
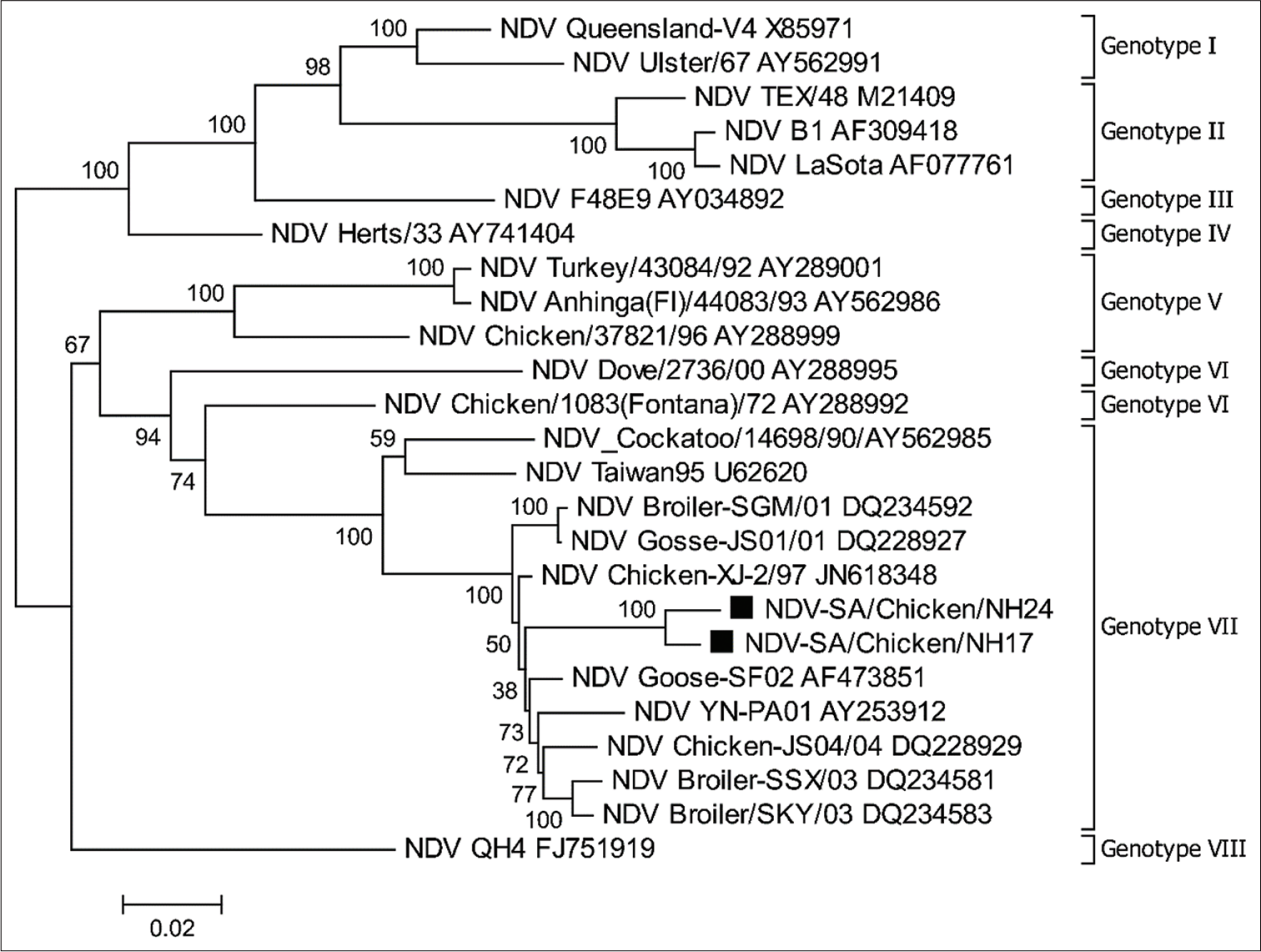
Phylogenetic analysis of complete hemagglutinin-neuraminidase gene sequences. The present Saudi isolates are marked with black squares. Reference sequences end with their GenBank accession numbers.

HN gene-based phylogenic analysis of the two representative isolates (NDV-SA/Chicken/NH17 and NDV-SA/Chicken/NH24) showed that the isolates were related to each other with 98.2% nucleotide identity (Figure-2 and Table-5). Both sequences clustered in genotype VII. A BLAST search showed that Chinese strain XJ-2/97 (GB# JN618348) was the most closely related sequence in GenBank, with nucleotide identities of 96% and 96.4% with NDV-SA/Chicken/NH17 and NDV-SA/Chicken/NH24, respectively. Nucleotide comparison with sequences belonging to genotype VII revealed identities ranging between 91.5% and 96.4%. The nucleotide identities with sequences from the other genotypes were ≤88.3% (Table-5).

**Table 5:** HN gene nucleotide sequence identities of two Saudi isolates with reference sequences from NDV genotypes.

Sequence	NDV-SA/Chicken/NH24	NDV-SA/Chicken/NH17
NDV-SA/Chicken/NH24		0.982
NDV-SA/Chicken/NH17	0.982	
NDV-Chicken-XJ-2/97/JN618348.1 (VII)	0.964	0.96
NDV-Gosse-JS01/01/DQ228927.1 (VII)	0.956	0.952
NDV-Broiler-SGM/01/DQ234592.1 (VII)	0.956	0.952
NDV-Chicken-JS04/04/DQ228929.1 (VII)	0.953	0.949
NDV-Broiler/SKY/03/DQ234583.1 (VII)	0.953	0.949
NDV-Broiler-SSX/03/DQ234581.1 (VII)	0.951	0.947
NDV-Goose-SF02/AF473851.2 (VII)	0.958	0.955
NDV-YN-PA01/AY253912.1 (VII)	0.946	0.943
NDV-Taiwan95/U62620.1 (VII)	0.924	0.92
NDV-cockatoo/14698/90/AY562985.1 (VII)	0.918	0.915
NDV-Chicken/1083(Fontana)/72/AY288992.1 (VI)	0.883	0.881
NDV-chicken/37821/96/AY288999.1(V)	0.863	0.862
NDV-Herts/33/AY741404.1 (IV)	0.851	0.852
NDV-F48E9/AY034892.1 (III)	0.828	0.83
NDV-LaSota/AF077761.1 (II)	0.813	0.815
NDV-Ulster/67/AY562991.1 (I)	0.833	0.833
NDV-QH4/FJ751919.1 (VIII)	0.855	0.856

NDV=Newcastle disease virus, HN=Hemagglutinin-neuraminidase

There was an inconsistency regarding the characteristics of the NDV-SA/Chicken/NH24 isolate. Partial F gene phylogeny and FPCS motif indicated that this isolate belongs to genotype II. *In vivo* biotyping showed virulence comparable to that of genotype VII. This was further supported by the complete HN gene phylogeny that showed NDV-SA/Chicken/NH24 as a part of genotype VII. One possible explanation for this confusion is the presence of mixed infection with both virulent genotype VII and vaccine strain belonging to genotype II. In this regard, the genotype VII-NDV strain (NDV-SA/Chicken/NH17) was isolated from the same farm 20 days earlier, and a vaccine containing the B1 strain was applied 11 days before sampling. Alternatively, natural recombination could have occurred at a certain point in time between genotype VII-like and genotype II-like viruses. Similar situations were previously reported in China and Iran [20,35,38-40]. A complete genome sequence of a similar NDV isolate revealed that recombination had occurred at the N terminus of the F gene [39].

The present genotype VII isolate shares considerable nucleotide identity with similar strains from the surrounding region, for example, with SMV-8/13 and SMV-4/2012 (GB# KU201415; KU201419) from Iran and SDSG01/2011 (GB # JN400896) from China. The trade of poultry and poultry products would be a possible dissemination route for NDV. Alternatively, wild migratory birds might play a possible role in its dissemination; this scenario was proposed for NDV dissemination in nearby Southern Iran [41]. Wild birds have been considered a potential reservoir of NDV. Low virulent NDV was frequently isolated from wild waterfowl. However, there is little evidence to suggest a possible role of wild birds in the dissemination of virulent NDV [42-45].

Vaccination is the primary measure to control ND [32]. The presented data showed that circulating NDVs belong to genotype II and virulent genotype VII. Strains belonging to genotypes VII and VI have been circulating in Saudi Arabia since 2000 and the 1990s, respectively, or even earlier [12]. Whether such a situation necessitates revision of the used vaccines is a controversial question. It has been well documented that NDV is a single serotype and that any strain can confer cross-protection against other strains. Recently, there has been a debate on whether some vaccine strains provide better protection than others depending on the titers of shed virus after challenge [1,46]. However, other investigators attribute the variations in virus shedding and even the occurrence of NDV outbreaks to inadequate administration of NDV vaccines [1].

## Conclusion

*In vivo* biotyping was used to confirm the results of molecular investigations. There were at least two NDV genotypes circulating in the Eastern Region of Saudi Arabia, genotype II and genotype VII. Mixed infection and possible natural recombination between the two genotypes must be considered. Surveillance of poultry respiratory diseases should be continued to optimize the control strategy. The role of wild migratory birds and semi-domesticated birds in the dissemination of NDV and other poultry respiratory pathogens need to be investigated.

## Authors’ Contributions

AIAA conceived and designed the study. The author collected the samples and analyzed the data. The author prepared and approved the final manuscript.
